# Valley phonons and exciton complexes in a monolayer semiconductor

**DOI:** 10.1038/s41467-020-14472-0

**Published:** 2020-01-30

**Authors:** Minhao He, Pasqual Rivera, Dinh Van Tuan, Nathan P. Wilson, Min Yang, Takashi Taniguchi, Kenji Watanabe, Jiaqiang Yan, David G. Mandrus, Hongyi Yu, Hanan Dery, Wang Yao, Xiaodong Xu

**Affiliations:** 1 Department of Physics, University of Washington, Seattle, WA 98195 USA; 2 Department of Electrical and Computer Engineering, University of Rochester, Rochester, NY 14627 USA; 3 National Institute for Materials Science, Tsukuba, Ibaraki 305-0044 Japan; 4 Materials Science and Technology Division, Oak Ridge National Laboratory, Oak Ridge, TN 37831 USA; 5 Department of Materials Science and Engineering, University of Tennessee, Knoxville, TN 37996 USA; 6 Department of Physics and Astronomy, University of Tennessee, Knoxville, TN 37996 USA; 7 Department of Physics and Center of Theoretical and Computational Physics, University of Hong Kong, Hong Kong, China; 8 Department of Physics and Astronomy, University of Rochester, Rochester, NY 14627 USA; 9 Department of Materials Science and Engineering, University of Washington, Seattle, WA 98195 USA

**Keywords:** Two-dimensional materials, Two-dimensional materials

## Abstract

The coupling between spin, charge, and lattice degrees of freedom plays an important role in a wide range of fundamental phenomena. Monolayer semiconducting transitional metal dichalcogenides have emerged as an outstanding platform for studying these coupling effects. Here, we report the observation of multiple valley phonons – phonons with momentum vectors pointing to the corners of the hexagonal Brillouin zone – and the resulting exciton complexes in the monolayer semiconductor WSe_2_. We find that these valley phonons lead to efficient intervalley scattering of quasi particles in both exciton formation and relaxation. This leads to a series of photoluminescence peaks as valley phonon replicas of dark trions. Using identified valley phonons, we also uncover an intervalley exciton near charge neutrality. Our work not only identifies a number of previously unknown 2D excitonic species, but also shows that monolayer WSe_2_ is a prime candidate for studying interactions between spin, pseudospin, and zone-edge phonons.

## Abstract (web summary)

In monolayer semiconductors phonons with momentum vectors pointing to the corners of the hexagonal Brillouin zone couple strongly to carriers’ spin and valley degree of freedom. Here, the authors report the observation of multiple valley phonons and the resulting exciton complexes in the monolayer semiconductor WSe_2_.

## Introduction

Electron–phonon interaction is a ubiquitous process in solids. In monolayer semiconducting transition metal dichalcogenides (TMDs), the broken inversion symmetry and strong spin–orbit coupling leads to the well-known spin–valley coupling of band edge electrons^1–6^. The emergent valley-contrasting properties not only impact quasiparticles, but also are expected to give rise to new physics involving zone edge phonons, or valley phonons, which are collective lattice oscillations at the corners of hexagonal Brillouin zone (±*K* points). These phonons have been predicted to play an important role in spin and valley pseudospin relaxation through phonon-assisted intervalley scattering^7,8^. Additionally, valley phonons can possess chirality with intrinsic pseudo-angular momentum^9^, which has recently attracted wide attention^10–13^. Such chiral phonons have nontrivial Berry curvature and are predicted to give rise to valley phonon Hall effect, a counterpart of valley Hall effect of electrons in 2D semiconductors^14–16^. Despite the importance of valley phonons, experimental progress in understanding their properties has been limited, since it is challenging to probe phonons with large momentum vectors.

Here, we identify the signatures of multiple valley phonons in monolayer WSe_2_. The monolayer WSe_2_ hosts stable and long-lived dark exciton and trion as ground states^17–22^. The resulting accumulated exciton and trion populations are highly desirable for studying their interactions with phonons. We found that three valley phonons facilitate efficient spin-conserving intervalley scattering, which results in a series of dark exciton and dark trion phonon-replicas in the low-temperature photoluminescence (PL) spectrum. The sign and magnitude of Landé effective *g*-factors of the various replicas, together with their PL helicity under optical pumping, reveals that the spin-preserving intervalley scattering of the electron is more efficient than its intravalley spin-flip during the dark exciton/trion formation process. This results in a surprising finding: the single electron in both the positive dark trion and intervalley exciton resides in the valley opposite to that which the optical pump is coupled to. Moreover, the identified intervalley exciton resonance enables us to infer a short-range electron–hole exchange interaction of ~10 meV by extracting the energy splitting between intervalley and dark (intravalley) excitons.

## Results

### Gate-dependent PL spectrum of monolayer WSe_2_

**Fig. 1:**
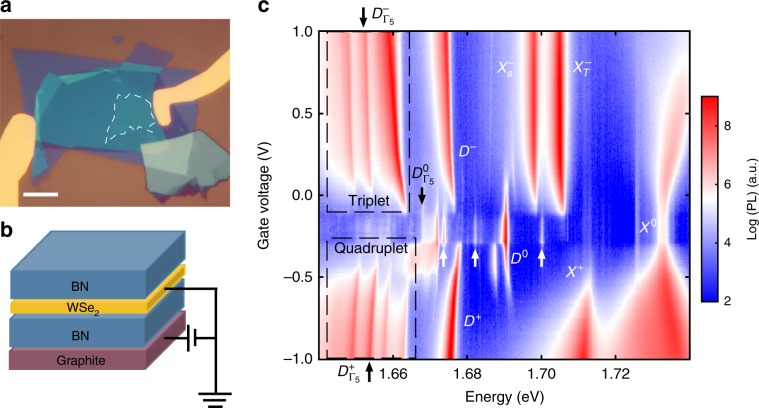
Gate-dependent photoluminescence. **a** Optical image of a representative gated WSe_2_ device, in which monolayer WSe_2_ (white dashed line area) is encapsulated in hBN with a graphite local back gate, scale bar is 10 µm. **b** Schematic of a gated WSe_2_ device. **c** Photoluminescence (PL) as a function of back gate voltage and photon energy. Excitonic states which have been reported in the literature are identified and marked. *X*^0^: neutral exciton; *X*^+^: positive trion; $X_{\mathrm{{S}}}^ -$ and $X_{\mathrm{{T}}}^ -$ : intravalley and intervalley trion; *D*^0^: spin forbidden neutral dark exciton; *D*^*+*^: positive dark trion; *D*^−^ negative dark trion. The unidentified triplet PL peaks at electron doping, quadruplet PL peaks at hole doping, and three states at the neutral regime, pointed to by the white arrows, are the focus of this work.

The samples are exfoliated monolayer WSe_2_ encapsulated between thin flakes of hexagonal boron nitride (hBN). Few-layered graphene serves as a local bottom gate for electrostatic control of the monolayer carrier density (see the “Methods” section). Figure 1a, b are an optical microscope image and schematic of a representative device, respectively. Figure 1c shows the PL intensity plot as a function of gate voltage (*V*) and photon energy, at a temperature of 1.6 K. The laser energy is 1.775 eV with right circularly polarized (*σ*^+^) excitation and unpolarized detection. The full helicity-resolved gate-dependent spectra are shown in Supplementary Fig. 1. Monolayer WSe_2_ hosts a rich spectrum of excitonic species^23–28^. Several previously identified excitonic states are indicated in the figure, including the neutral bright exciton, bright trions^29–33^, the intravalley spin-forbidden dark exciton^17–20,34^ and dark trions^18,35^. The recently identified zone-center Γ_5_—or *E*''—phonon replicas below both neutral^12,13^ and charged dark excitons^12^ are also resolved, as indicated by black arrows.

The focus of this paper is to understand the valley phonon origin of several previously unidentified PL peaks. In particular, we focus on the triplet and quadruplet PL peaks outlined by dashed black boxes, and those pointed at by the white arrows in Fig. 1c. We have measured multiple samples that exhibit similar spectrum (Supplementary Fig. 2), and the nearly identical power dependence of the peaks of interest across several samples rules out the possibility of them arising due to defect states (Supplementary Fig. 3). As we explain below, all these peaks arise from the coupling of optically dark states to the bright ones via emitting valley phonons, which results in a series Stokes-shifted valley phonon-replicas of the dark exciton and dark trions in the PL spectrum.

### Valley phonon replicas of positively charged dark trion

**Fig. 2:**
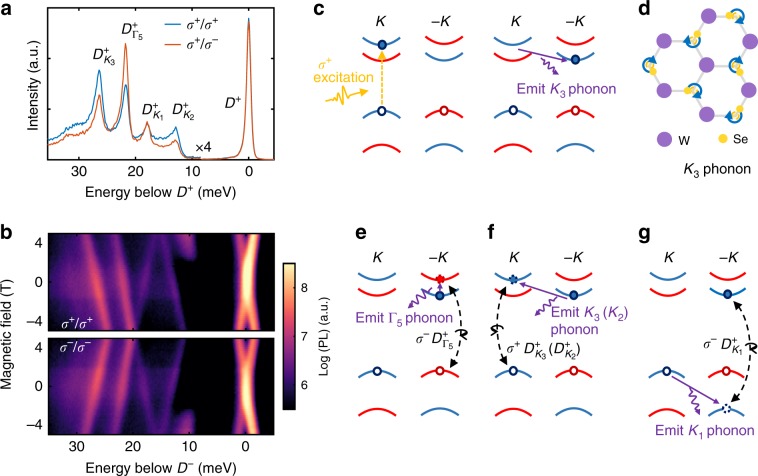
Valley phonon replicas of positively charged dark trion. **a** Circular polarization resolved PL of the quadruplet PL peaks on the hole doping side. The photon energy of the spectrum is offset with respect to the positively charged dark trion (*D*^+^). The intensity of the quadruplets is multiplied by a factor 4 to emphasize the weak spetrum features. **b** Circular polarization resolved magneto PL of the quadruplets with *σ*^+^/*σ*^+^ (top) and *σ*^−^/*σ*^−^ (bottom) excitation/ detection. **c** Schematic of *D*^+^ formation process under *σ*^+^ excitation. Blue and red represent bands with electron having spin up and down, respectively. Filled and unfilled circles represent electron and missing electron (hole) in the conduction and valence band, respectively. Orange dashed line indicates photo excitation. Purple arrow denotes the emission of valley phonon. **d** Illustration of vibrational normal mode of *K*_3_ phonon, wherein Se atoms orbit around their equilibrium positions. See Supplementary Fig. 6 for other valley phonons. **e**–**g** Schematic of phonon-assisted emission process of $D_{{\mathrm{\Gamma }}_5}^ +$, $D_{{\mathrm{K}}_3}^ + \left( {D_{{\mathrm{K}}_2}^ + } \right)$, and $D_{{\mathrm{K}}_1}^ +$phonon replicas, respectively. Dashed circle: virtual state. As indicated by the purple arrow, the optically dark state couples to the bright ones by emitting a valley phonon, resulting in a Stokes shift of the dark trion emission by the recombination of an electron–hole pair (black dashed line). See text for details.

We first consider the quadruplet PL peaks in the hole doping regime. Figure 2a shows the circular polarization resolved PL with *σ*^+^ polarized excitation. The photon energy is relative to the positive dark trion (*D*^+^), and the intensity of the quadruplets is multiplied by a factor of 4 to emphasize these weak spectral features. We ascribe the four peaks to bright replicas of the dark trion, mediated by interactions with phonons. The quadruplets are labeled as $D_{K_3}^ +$, $D_{{\mathrm{\Gamma }}_5}^ +$, $D_{K_1}^ +$, $D_{K_2}^ +$, which correspond to their origin from coupling of *D*^+^ with valley phonons *K*_3_, *K*_1_, and *K*_2_, and the zone-center phonon Γ_5_. This nomenclature has its origin in the Koster notation of the *K*-point irreducible representations of the *C*_3*h*_ point double-group corresponding to the symmetry of the monolayer semiconductors (Supplementary Table 3, character table).

The valley phonon replicas $D_{K_3}^ + ,D_{K_1}^ + ,D_{K_2}^ +$ are 26, 18, and 13 meV below *D*^+^, respectively. The association with zone-edge phonons *K*_3_, *K*_1_, and *K*_2_ is two-fold. The first reason is that the energy differences between *D*^+^ and its replicas match the energies of the associated phonon modes (Supplementary Fig. 5, phonon spectrum). The second reason is rooted in the selection rules of electron–phonon coupling;^7^ intervalley transitions of electrons and holes are mediated only by specific phonons (see discussions in Supplementary Notes 3 and 4). Finally, we note that $D_{{\mathrm{\Gamma }}_5}^ +$ appears 22 meV below *D*^+^, which is consistent with the recently reported Γ_5_ phonon replica of *D*^+^^12^.

To further examine these assignments, we performed magneto-PL to extract the effective Landé *g*-factors of the various states, which are then used to identify the spin and valley indices of the constituent electrons and holes in the excitonic states^36–40^. The extracted *g*-factors of the states of interest are listed in Table 1. The *g*-factors of other states are listed in Supplementary Table 1, and a detailed analysis can be found in Supplementary Note 2. Briefly, for intravalley electron–hole recombination, the magnitude of the *g*-factor is about 4 for parallel electron and hole spins^36,37,39,40^, and about 9 when the two spins are anti-parallel^12,13,21^. Meanwhile, if the recombination involves electron and hole from opposite valleys but with parallel spins, a value about 13 is expected.

**Table 1:** Effective Landé *g*-factors of WSe_2_ dark excitonic states and their phonon replicas.

	Dark state	*K*_2_ replica	*K*_1_ replica	Γ_5_ replica	*K*_3_ replica
Neutral regime: *I*^0^	−12.5	–	12.0	–	−12.6
Neutral regime: *D*^0^	−9.1	–	–	−9.8	–
Hole doped: *D*^+^	−8.6	−13.0	12.2	−9.7	−13.4
Electron doped: *D*^−^	−9.5	−13.6	12.2	−9.9	−12.5

Figure 2b shows the circular polarization resolved PL intensity as a function of out-of-plane magnetic field. The energy scale is relative to the position of *D*^+^ at zero applied magnetic field. Both the cross pattern of *D*^+^ in Fig. 2b and its unpolarized light emission (see Fig. 2a) result from the underlying out-of-plane dipole orientation^12^, and are hallmarks of direct intravalley recombination of dark trions. Following the convention of valley Zeeman splitting as *Δ* = *E*(*σ*^+^)−*E*(*σ*^−^), where *E*(*σ*^+^) and *E*(*σ*^−^) are the peak energies of the *σ*^+^ and *σ*^−^ polarized PL components, we obtained *g*(*D*^+^) = −8.6. The obtained *g*-factor of *D*^+^ is therefore consistent with expectations for the direct recombination through the intravalley spin–flip transition.

The Zeeman shifts of the quadruplet PL peaks underpin their origin as phonon replicas of the dark positive trion *D*^+^. The extracted *g*-factors of −13.4, 12.2, and −13.0 for $D_{K_3}^ +$, $D_{K_1}^ +$, $D_{K_2}^ +$, respectively, are consistent with intervalley recombination of electron and hole. Note that the *g* factor sign of $D_{K_1}^ +$ is opposite to others, which will be discussed later. While the intervalley recombination is naturally forbidden for delocalized exciton complexes, because of the large momentum mismatch, emission of a valley phonon can supply the required momentum, resulting in phonon-assisted luminescence of the otherwise dark states. Combined with the concurrence of similar valley phonon energies and energy difference between the peaks and $D^ +$, we conclude that $D_{K_3}^ +$, $D_{K_1}^ +$, $D_{K_2}^ +$ are *K*_3_, *K*_1_, and *K*_2_ valley phonon replicas of *D*^+^.

The polarization of the $D_{{\mathrm{\Gamma }}_5}^ +$ peak highlights the importance of valley phonons in the formation process of *D*^+^. From Fig. 2a, we observe that $D_{{\mathrm{\Gamma }}_5}^ +$ is cross-circularly polarized. The valley optical selection rules dictate that *σ*^+^ excitation creates an electron and hole in the +*K* valley, while *σ*^−^ polarized emission can only happen through spin-conserved electron–hole recombination in the −*K* valley. In addition, $g\left( {D_{{\mathrm{\Gamma }}_5}^ + } \right)$ = −9.7 indicates the intravalley electron–hole recombination nature of the peak. The cross-polarized emission of $D_{{\mathrm{\Gamma }}_5}^ +$ therefore leads to the surprising conclusion that *σ*^+^ excitation results in *D*^+^ with the single electron located in the −*K* valley: i.e. *D*^+^(−*K*).

The cross polarization of *D*^+^ can be understood by considering the impact of phonons on the electron relaxation pathways following photoexcitation. The *D*^+^ population is created by optical pumping of the spin-conserved interband transition (Fig. 2c), followed by the relaxation of the electron from the higher energy spin-valley locked sub-band to the lower energy one. The latter step requires either a spin–flip or a valley–flip. From symmetry analysis, the Γ_5_ phonon can lead to the intravalley spin–flip relaxation of electron from upper to lower conduction band^7^, but it cannot cause intervalley scattering. On the other hand, spin-conserving intervalley scattering of the electron with *K*_3_ phonon is a symmetry-allowed zeroth-order channel^8^. Figure 2c illustrates the intervalley electron–phonon relaxation process that leads to the formation of *D*^+^ (−*K*). First, *σ*^+^ excitation creates electron in the spin up conduction band in the *K* valley. Assisted by the *K*_3_ valley phonon (Fig. 2d), this spin up electron is then scattered into the spin up band in the −*K* valley, forming *D*^+^(−*K*) with two holes separately located at the top of ±*K* valleys. *D*^+^(−*K*) then couples to *σ*^−^ polarized photon by emitting Γ_5_ phonon, as shown in Fig. 2e. Evidently, the observed cross-polarized $D_{{\mathrm{\Gamma }}_5}^ +$ emission implies that the valley–flip rate exceeds the spin–flip one in the relaxation of electron.

Having established that *σ*^+^ polarized excitation results in *D*^*+*^(−*K*), the understanding of both $D_{K_3}^ +$ and $D_{K_2}^ +$ is straightforward. As indicated in Fig. 2f, the spin up electron in the lower −*K* sub-band is virtually scattered to the higher +*K* sub-band by emitting either a *K*_3_ or *K*_2_ valley phonon (see Supplementary Note 5 for further analysis of *K*_2_). The spin-conserving intervalley scatter then allows for recombination with the hole in the +*K* valley, emitting *σ*^+^ polarized photon with energy either 26 meV (*K*_3_) or 13 meV (*K*_2_) below D^+^. The *g*-factors of $D_{K_3}^ +$ and $D_{K_2}^ +$ are nearly equal, about −13.4 and −13.0, respectively, and correspond to expected values for the intervalley spin-conserving electron–hole recombination.

Moreover, the measured amplitude of $D_{K_3}^ +$, which is several times stronger than that of $D_{K_2}^ +$, is also consistent with group-theory selection rules. In particular, the selection rules dictate that, in pristine monolayer WSe_2_, only the *K*_3_ phonon mode can induce intervalley electron transitions between conduction band edges at the high symmetry *K* and −*K* points^7^. Meanwhile, intervalley electron transitions that are mediated by other *K*-point phonon modes are higher-order processes with correspondingly smaller amplitudes that involve electron states in the neighborhood of ±*K*. Such relatively weak processes can be amplified by several possible sources, such as localization next to defects, breaking WSe_2_ mirror inversion symmetry by hBN encapsulation, or WSe_2_/hBN moire superlattice providing in plane momentum.

The positive *g*-factor of $D_{{\mathrm{K}}_1}^ +$ is a signature of the interaction of the hole and *K*_1_ valley phonon. According to the group-theory selection rules, *K*_1_ is the only phonon mode that enables the spin-conserving intervalley transition between valence-band states at *K* and −*K* points (i.e., it is a zeroth-order process)^7^. As shown in Fig. 2g, the hole in the higher valence band of *K* valley is virtually scattered to the lower valence band of the −*K* valley with the same spin orientation via emission of a *K*_1_ valley phonon, forming an intermediate virtual B trion. The recombination of the electron with this scattered hole in the −*K* valley results in $D_{K_1}^ +$, with energy 18 meV below *D*^+^. While the initial and final states have the same spin-valley configuration as in $D_{K_3}^ +$ and $D_{K_2}^ +$, the emission from $D_{K_1}^ +$ is *σ*^−^ polarized. Therefore, the *g*-factor of $D_{K_1}^ +$ is expected to have similar magnitude, but opposite sign, compared to the *g*-factors of $D_{K_3}^ +$ and $D_{K_2}^ +$, as we have observed. Note that although the interaction between the hole and valley phonon *K*_1_ is relatively strong, the coupling of *D*^*+*^(−*K*) (or A trion) with the virtual B trion state is ~400 meV detuned, which is much larger than in the case of *K*_3_ intervalley electron scattering. Therefore, the PL intensity of $D_{K_1}^ +$ is expected to be several times weaker than that of $D_{K_3}^ +$, which is in agreement with our observation (see Supplementary Note 4).

### Valley phonon replicas of negatively charged dark trion

**Fig. 3:**
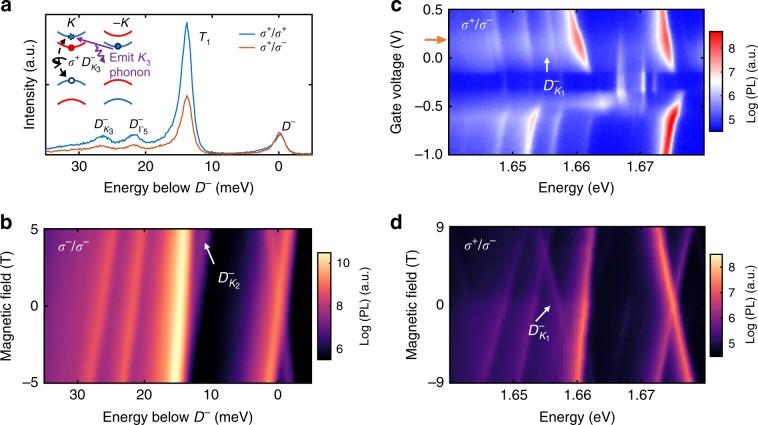
Valley phonon replicas of negatively charged dark trion. **a** Circular polarization resolved PL of the triplet PL peaks under electron-doped conditions. The photon energy of the spectrum is offset with respect to the negatively charged dark trion (*D*^−^). **b** Magneto PL of the triplets with *σ*^−^ excitation and co-polarized detection. The photon energy of the spectrum is offset with respect to *D*^−^ at zero field. **c** Gate-dependent PL with *σ*^+^ resonant pumping of the bright exciton and cross polarized (*σ*^−^) detection. The $D_{K_1}^ -$ state on the electron doping side, which is 18 meV below *D*^−^, is clearly resolved. **d** Magneto PL at the gate voltage indicated by the orange arrow in **c**, with σ^+^ excitation and *σ*^−^detection.

The valley phonon replicas of the negative dark trion *D*^−^ can be understood using similar analysis as above. Figure 3a shows the helicity-resolved PL under *σ*^+^ polarized excitation. The lower energy spectral features, denoted as *T*_1_, $D_{K_3}^ -$, and $D_{{\mathrm{\Gamma }}_5}^ -$ show appreciable co-circular polarization. Figure 3b shows the PL intensity as a function of magnetic field and photon energy, with *σ*^−^/*σ*^−^ polarized excitation/detection (the *σ*^+^/*σ*^+^ results are shown in Supplementary Fig. 7). The extracted *g*-factor of $D_{K_3}^ -$ is −12.5, indicating intervalley recombination, and the energy is 26 meV below *D*^−^. As such, we can identify that $D_{K_3}^ -$ originates from the interaction of −*K* valley electron with *K*_3_ phonon, as depicted in the inset of Fig. 3a. The *g*-factor of $D_{{\mathrm{\Gamma }}_5}^ -$ (−9.9) is nearly the same as that of *D*^−^ (−9.5), and as expected, the peak appears 22 meV below *D*^−^.

The observation of triplet peak pattern in the PL spectrum under electron doping is slightly different from the quadruplet one observed under hole doping. Since *T*_1_ is more intense than *D*^−^, and its *g*-factor is about −4.5, it is not likely to be a phonon replica of *D*^−^. Its origin is unknown, but *T*_1_ is near the spectral range of the *K*_2_ and *K*_1_ valley phonon replicas, and thus obscures them entirely. Nevertheless, the large difference in *g*-factor of *T*_1_ from that of *D*^−^ allows us to identify $D_{K_2}^ -$ in magneto-PL, since the states shift away from one another. We note that there is a faint line in Fig. 3b, appearing on the high energy shoulder of *T*_1_ at high magnetic field. We ascribe this peak to $D_{K_2}^ -$ based on its *g*-factor of about −13.6, and its energy difference from *D*^−^ of 13 meV, both of which are in good agreement with expectations for *K*_2_ phonon replica.

Meanwhile, $D_{K_1}^ -$ becomes evident when the excitation laser is resonant with the bright neutral exciton (*X*^0^). Figure 3c presents the PL intensity as a function of gate voltage and photon energy, with *σ*^+^ polarized excitation laser in resonance with *X*^0^ (1.733 eV) and *σ*^−^ polarized detection. For *V* = 0.2 V, arrow on side of Fig. 3c, $D_{K_1}^ -$ becomes apparent at 18 meV below *D*^−^. Figure 3d shows magneto-PL with the same cross-polarized polarization (*σ*^+^/*σ*^−^) (see Supplementary Fig. 8 for complete data set). We find that $D_{K_1}^ -$ has a positive *g*-factor of 12.2, which is identical to that of $D_{K_1}^ +$. Therefore, despite the initial appearance, the four phonon replica states observed under hole-doping also appear under electron-doping conditions. Moreover, we observe similar magnitude and sign of *g*-factors, as well as energy separation from the parent dark trion state.

### Identification of intervalley dark excitons and its valley phonon replica

**Fig. 4:**
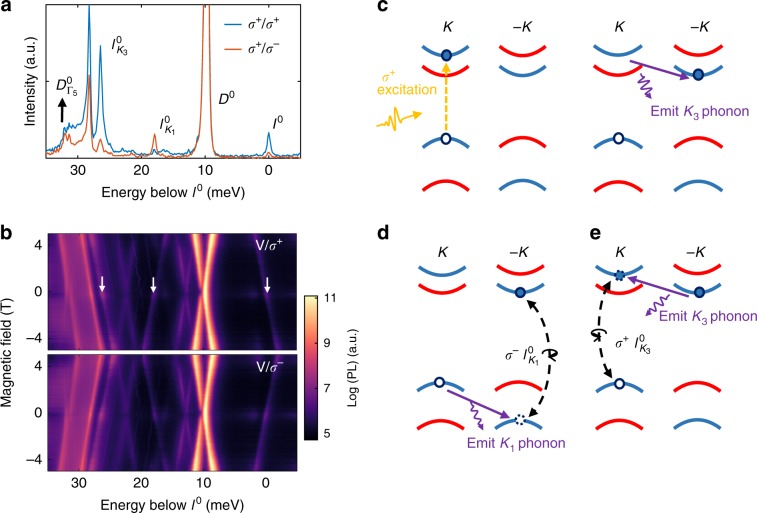
Identification of intervalley dark exciton and its valley phonon replicas. **a** Circular polarization resolved PL in the neutral regime. The photon energy of the spectrum is offset with respect to the intervalley exciton (*I*^0^). **b** Magneto PL with linearly polarized excitation, *σ*^+^ (up) and *σ*^−^ polarized (down) detection. $I_{K_3}^0$, $I_{K_1}^0$ and *I*^0^ are labeled by the white arrows, from left to right. **c** Schematic of intervalley exciton formation process under *σ*^+^ excitation, with assistance of *K*_3_ phonon. **d**, **e** Schematic of light emission process of $I_{K_1}^0$ (left) and $I_{K_3}^0$(right) phonon replicas.

The valley phonon-assisted momentum relaxation mechanism also produces phonon replicas for neutral dark excitons. Figure 4a shows the helicity-resolved PL spectra near charge neutrality (*V* = −0.2 V). The energy axis is offset from *I*^0^, which is the sharp line 32 meV below the bright neutral exciton in Fig. 1c. We also observe strong direct recombination from the neutral intravalley dark exciton *D*^0^, at 42 meV below *X*^0^. We confirm that *D*^0^ has negligible circular polarization, resulting from its out-of-plane dipole orientation, and the extracted *g*-factor (−9.1) is in good agreement with previously reported values^12,13,21^. Magneto-PL measurements with linear excitation (*V*) and *σ*^+^/*σ*^−^ collection are shown in the top/bottom panel of Fig. 4b, respectively. The observed PL peak at 22 meV below *D*^0^, with a *g*-factor of −9.8 is consistent with the reported Γ_5_ phonon replica $D_{{\mathrm{\Gamma }}_5}^0$^12,13^. We further note that $D_{{\mathrm{\Gamma }}_5}^0$ shows a zero-field splitting of 0.6 meV, which arises from the fine structure of *D*^0^ (Supplementary Fig. 9)^8,21^. We note that there are two replicas of *D*^0^ that are apparent in the magneto-PL (cross-patterns at 3 and 13 meV below *D*^0^). The peak position of the latter one matches that of positive dark trion thus is precursor of *D*^+^, while the former one cannot be unambiguously identified at this time.

The *I*^0^ emission is distinct from that of *D*^0^ and exhibits all the expected behavior of the momentum indirect, or intervalley dark exciton, which has not been previously identified in this system. This emission peak is strongly co-circularly polarized, with near unity polarization. Its *g*-factor of about −12.5 corresponds to the recombination of electron and hole residing in opposite valleys, implying that *I*^0^ is the direct recombination of intervalley exciton. The formation of *I*^0^ is a consequence of the fast valley-flip of electrons via scattering with *K*_3_ phonon in the dark exciton formation, as illustrated in Fig. 4c. This is consistent with formation of *D*^+^(−*K*) discussed above (Fig. 2c). The 10 meV energy splitting between *D*^0^ and *I*^0^ is then a direct measure of the short-range electron–hole exchange interactions^41^. We note that the direct photon emission from the momentum indirect *I*^0^ is weak in intensity, even when compared to the spin-forbidden dark state *D*^0^. However, weak PL from indirect states is not unprecedented. Similar to the case of indirect band-gap semiconductors such as silicon, recombination of intervalley excitons without phonons can be mediated by localization next to defects^42^, which alleviates the need to conserve crystal momentum due to translation symmetry.

The assignment of *I*^0^ is corroborated by the identifications of its *K*_1_ and *K*_3_ phonon replicas, made evident by examining the energy, polarization, and *g*-factors of the spectral features. The feature indicated as $I_{K_1}^0$ in Fig. 4a, is located 18 meV below *I*^0^, is cross-circularly polarized, and has a positive *g*-factor of 12.0. These values are nearly identical to those found for both $D_{K_1}^ +$ and $D_{K_1}^ -$, and consistent with expectations for the *K*_1_ valley phonon replica of *I*^0^. The $I_{K_1}^0$ recombination process via intervalley hole scattering is illustrated in Fig. 4d. In addition, the peak $I_{K_3}^0$ is about 26 meV below *I*^0^ and has a *g*-factor of −12.6, which is the same as *I*^0^ and supports its origin as *K*_3_ valley phonon replica of *I*^0^ (Fig. 4e).

## Discussion

In conclusion, we unravel the role of valley phonons in exciton and trion formation and their recombination in semiconducting monolayer WSe_2_. Our work settles questions of the origin of nearly all of the observed peaks in the complex excitonic spectrum of monolayer WSe_2_. Another important result is that the relaxation of optically generated electrons from the upper conduction band to the lower conduction band is dominated by *K*_3_ phonon-assisted spin-conserving intervalley scattering, rather than the Γ_5_ phonon-assisted spin–flip intravalley scattering. Such a relaxation pathway gives rise to the unexpected initial state for the positively charged dark trion, and efficient formation of intervalley exciton *I*^0^. This understanding is important for correct interpretation of excitonic spectral features, and may allow for new schemes to control the electron/exciton spin-valley state via optical pumping, e.g. coherent control of *D*^0^ and *I*^0^ populations via stimulated Raman adiabatic passage. Our work further motivates detailed studies of the electron-valley phonon coupling matrix elements by the first principle calculations, which should provide insights for theoretical models to gain a complete understanding of the complex monolayer WSe_2_ spectrum.

## Methods

### Sample fabrication

Monolayers of WSe_2_ were mechanically exfoliated from bulk crystals and identified by optical contrast, which was later confirmed by their low-temperature PL spectrum. Thickness of hBN flakes used for encapsulation was typically 10–20 nm, while the thickness of graphite back gate electrodes was typically around 5 nm. Heterostructures of hBN/WSe2/hBN/Graphite are made with dry-transfer technique using polycarbonate films^43^. The surface of every flake in the heterostructure was confirmed clean with atomic force microscopy prior to fabrication. Finally, the V/Au contact are patterned with standard electron beam lithography and evaporation.

### PL spectroscopy

PL measurements were performed with a confocal microscope in reflection geometry, with sample mounted in an exchange gas cooled cryostat (AttoDry 2100). The cryostat is equipped with a superconducting magnet in Faraday geometry (magnetic field *B* perpendicular to sample plane). All measurements were performed at 1.6 K unless otherwise specified. A He–Ne laser (632.8 nm) or a frequency tunable continuous-wave Ti:sapphire laser were used to excite the sample. Polarization resolved PL measurements were performed with a set of broad-band half-wave plates, quarter wave plates and linear polarizers. PL signal was collected by a spectrometer with a silicon charge-coupled device.

## Supplementary information

Supplementary Information

## Data Availability

The data that support the findings of this study are available from the corresponding authors upon reasonable request.
