# Supplementary material for: Valley phonons and exciton complexes in a monolayer semiconductor
Source: Nat Commun. 2020 Jan 30;11:618. doi: 10.1038/s41467-020-14472-0 (PMC6992782; doi:10.1038/s41467-020-14472-0)
Supplement: Supplementary file 1 — Supplementary Information [file 41467_2020_14472_MOESM1_ESM.pdf]

Supplementary Information for

Valley Phonons and Exciton Complexes in a Monolayer Semiconductor

Authors: Minhao He[1†], Pasqual Rivera[1†], Dinh Van Tuan[2], Nathan P. Wilson[1], Min Yang[2], Takashi Taniguchi[3], Kenji Watanabe[3], Jiaqiang Yan[4,5], David G. Mandrus[4-6], Hongyi Yu[7], Hanan Dery[2,8*], Wang Yao[7*], & Xiaodong Xu[1,9*]

Affiliations:

[1]Department of Physics, University of Washington, Seattle, Washington 98195, US A

[2]Department of Electrical and Computer Engineering, University of Rochester, Rochester, New York, 14627, USA

[3]National Institute for Materials Science, Tsukuba, Ibaraki 305-0044, Japan

[4]Materials Science and Technology Division, Oak Ridge National Laboratory, Oak Ridge, Tennessee, 37831, USA

[5]Department of Materials Science and Engineering, University of Tennessee, Knoxville, Tennessee, 37996, USA

[6]Department of Physics and Astronomy, University of Tennessee, Knoxville, Tennessee, 37996, USA

[7]Department of Physics and Center of Theoretical and Computational Physics, University of Hong Kong, Hong Kong, China

[8]Department of Physics and Astronomy, University of Rochester, Rochester, New York 14627, USA

[9]Department of Materials Science and Engineering, University of Washington, Seattle, Washington, 98195, USA

[†] These authors contributed equally to the work.

[*]Correspondence to xuxd@uw.edu; wangyao@hku.hk; hanan.dery@rochester.edu

Supplementary Note1. Discussion of possible defect localization effect.

Here we discuss the experimental evidences which rule out the observed valley phonon replicas as states localized to defects. Supplementary Figure 3 shows the power dependence of the observed states. The power dependence of the phonon replicas is the same as their associated dark state, which supports their valley phonon origin. In specific, the power dependence of the phonon replicas of negative dark trion (D^-) is linear (Supplementary Figure 3d). Localized states usually show sublinear behavior due to the long lifetime. Sublinear power dependence is indeed observed for the replicas in the neutral and positive charged regime. However, here the sublinear effects are from the long lifetime of the optically dark states (D^+, D^0, and I^0). Using replicas associated with positively dark trion (D^+) as example, D^+ photoluminescence intensity has sublinear power dependence $I \sim P^{\alpha}$ with α=0.9 (Supplementary Figure 3f), which results from the long lifetime of D^+. The PL intensity of the four lower energy peaks, all have same sublinear power dependence with α=0.9 as D^+. This is consistent with all four peaks as valley phonon replica of D^+.

More importantly, these phonon replica features (10 spectral peaks) are highly repeatable from sample to sample in terms of the spectral structure (Supplementary Figure 2), g factor, polarization, and their relative spectral energies. In fact, the spectral structure is the same for both positive and negative trions, as we demonstrated. Such repeatable and robust properties are distinct from the random spectral features localized to defects. We emphasize that all spectral features can be well explained by the valley phonon assisted intervalley scattering of electrons or holes. It is worth pointing out the sign of the g factor as well as the sign of PL polarization are different between different states, which are also consistent with different nature of the valley phonons involved in intervalley quasiparticle relaxation. The evidence of these states as valley phonon replicas, rather than defect bound states, are overwhelming.

Supplementary Note2. Landé effective g-factor analysis.

In monolayer WSe_2, Zeeman splitting of excitonic states at the $\pm K$-point valleys can be attributed to three main contributions[1-4]. The first part comes from spin of the composite quasiparticles in the excitonic state, which gives a Zeeman energy of $2S_z\mu_B B$. The second part comes from the atomic orbital magnetic moment, giving a Zeeman energy of $m\mu_B B$. In the case of monolayer WSe2, the conduction band edge is mainly composed of d-orbitals with m = 0 in both K-point valleys, whereas the valence band edges in the K($-K$) point valley are mainly d-orbitals with $m = 2(-2)$ in the upper valence band, and $m = -2(2)$ in the lower valence band. Lastly, it is shown that the lattice structure also contributes a valley magnetic moment with a g-factor of $\alpha_c(\alpha_v)$ for the conduction(valence) band, resulting in a Zeeman energy of $\alpha_c(\alpha_v)\ \mu_B B$ for an electron (hole), respectively.

Supplementary Figure 4a shows three main contributions to the Zeeman shift at the band edges in $\pm K$ point valleys. We identify three representative spin-valley configurations involved in the light emission process of all the excitonic states in Supplementary Figure 1a. Notice that for the trion states, the quasiparticle that remain unchanged after light emission does not affect the measured Zeeman splitting. Thus, we only consider spin-valley configuration of the electron-hole pair involved in the recombination. Intravalley bright exciton recombination, shown in

Supplementary Figure 4b, refers to recombination of an electron in the conduction band and a missing electron (hole) in the valence band with the same spin and valley quantum numbers. The calculated effective g-factor is $2(\alpha_c-\alpha_v-2)$, and is experimentally measured to be about -4. This means the valley magnetic moments for the conduction and valence bands are almost the same, which agrees well with theory[3-6].

Intravalley dark recombination refers to recombination of an electron and a missing electron (hole) in the same valley with opposite spin quantum number, as shown in Supplementary Figure 4c. Similarly, we can calculate that the effective g-factor is $2(\alpha_c-\alpha_v-4)$. The corresponding D^0, D^+ and D^- states have an effective g-factor close to -9[7-9]. Finally, the intervalley recombination refers to recombination of an electron and a missing electron (hole) in the opposite valleys, but with the same spin quantum number. The effective g-factor can be written as $2(-\alpha_c-\alpha_v-4)$ for K_3 and K_2 valley phonon assisted intervalley recombination. It has the same amplitude but opposite sign for K_1 valley phonon assisted intervalley recombination. The complication of the sign of g-factors comes from the circular polarization of the emitted photon, as a result of it being either an intervalley electron scattering process or intervalley hole scattering process. The magnitude of the effective g-factor for the intervalley recombination is measured be about 13.

In Supplementary Table 1, we summarized the measured effective g-factor of the excitonic states identified in Supplementary Figure 1a. We extract the σ^+ and σ^- polarized PL peak energy, $E(\sigma^+)$ and $E(\sigma^-)$, respectively. The effective g-factor is then calculated from the extracted Zeeman splitting: $\Delta = E(\sigma^+) - E(\sigma^-)$.

Supplementary Note3. Selection rules for intervalley transitions.

Phonon-induced intravalley transitions between bright and dark excitons

Supplementary Table 2 shows the character table of D_{3h} point double-group[10]. This table is used to derive selection rules for phonon-mediated transitions of intravalley excitons, corresponding to the case that both electron and hole reside in the same valley (e.g., both in the *K*-point valley)[11]. The transformation properties of optically-inactive dark excitons are captured by the irreducible representation (IR) Γ_3, semi-dark excitons with out-of-plane optical transition dipole by Γ_4, and bright excitons with in-plane optical transition dipole by Γ_6[12]. From Supplementary Table 2, one can readily verify the selection rule,

$$(\Gamma_6^* \times \Gamma_3)^* = (\Gamma_6^* \times \Gamma_4)^* = \Gamma_5, \quad \text{(S1)}$$

implying that transitions between dark and bright excitons due to intravalley spin flip (of the electron component) can be induced by a zone-center phonon that transforms like the IR Γ_5. This phonon is the homopolar optical mode, whose polarization vector is denoted by in-plane and out-of-phase vibration of the chalcogen atoms, as shown in Supplementary Figure 5a[12]. Note that the spin-flip matrix element due to interaction with a long-wavelength flexural phonon, which transforms as Γ_4, is nonzero only if the phonon wavevector is finite (i.e., when $q \neq 0$)[11]. On the other hand, the phonon mode Γ_5 is the only one for which the spin-flip matrix element is nonzero exactly at the zone center ($q = 0$).

Zone-edge phonons

Using Quantum Expresso[13], Supplementary Figure 5b shows the calculated phonon spectrum in monolayer WSe_2. Because the concept of acoustic and optical phonon modes near the Γ-point

loses its meaning when qa is no longer much smaller than unity (a is the lattice constant), the notion of acoustic or optical phonons for longitudinal and transverse modes (i.e., LA, TA, LO and TO) is not valid when dealing with zone-edge phonons at the K-point whose wavenumber is $q = K = 4\pi/3a$. Instead, the use of K-point IRs is more appropriate. This nomenclature is presented in Supplementary Table 3, where the transformation properties of the zone-edge phonons are captured by the IRs K_1 through K_6 (these IRs also belong to the C_{3h} point single group. The spin quantum number has no bearing on the polarization vectors of atoms in the unit cell)[11].

The nine polarization vectors of the zone-edge phonons are presented in the last column of Supplementary Table 3. $M_{z/||}(x,y,z)$ and $X_{\pm,z/||}(x,y,z)$ are polarization vectors for the transition-metal and chalcogen atoms, respectively. The subscripts z and || denote out-of plane (z) and in-plane (||) vibrations. The subscript '+(−)' of X denotes in(counter)-phase motion of the two chalcogen atoms in a unit cell. Supplementary Table 3 shows that each of the IRs K_{3-5} is associated with one polarization vector, and consequently, each of these IRs represents one zone-edge phonon. The IRs K_1, K_2 and K_6 are different in that each contains two types of atomic displacements, and thus, each corresponds to two independent zone-edge phonon modes. From energy considerations that will be explained below and using Supplementary Figure 5b as a typical example for ML-TMDs, K_{3-5} represent zone-edge phonons from the mid-three bundle whereas the bottom (top) three zone-edge phonons are associated with the low- (high-) energy modes of K_1, K_2 and K_6. The atomic displacement that corresponds to the mode K_l with energy $\hbar\omega_{Kl}$ is proportional to

$$\delta R^{K_1}_{\alpha,j} \propto V_\alpha(K_1)e^{i\left(K\cdot R_{\alpha,j}+\omega_{K_1}t\right)} + V^*_\alpha(K_1)e^{-i\left(K\cdot R_{\alpha,j}+\omega_{K_1}t\right)}. \qquad \text{(S2)}$$

$R_{\alpha,j}$ is a 2D vector denoting the equilibrium position of the α-atom in the jth unit cell, where $\alpha = (M,X_t,X_b)$ and $X_{t/b}$ denotes the top/bottom chalcogen atoms. $V_\alpha(K_l)$ is the polarization vector (right column of Supplementary Table 3). Given that $|K| = 4\pi/3a$ where a is the in-plane distance between nearby identical atoms, the phase difference obeys $K\cdot(R_{X(M),j} - R_{X(M),j+n}) = \pm 2\pi n/3$, where n is the number of unit cells along a zig-zag chain. Consequently, the displacement of every third chalcogen (or transition-metal) atom along a zigzag chain is identical. In addition, when substituting the in-plane polarization vectors $V_\alpha = [1,\pm i,0]$ in Eq. (S2), the resulting atomic motion follows a clockwise or counterclockwise circular trajectory, $\delta R^{K_1}_{\alpha,\mathrm{j}} \propto \cos\left(K\cdot R_{\alpha,\mathrm{j}} + \omega_{K_1}\mathrm{t}\right)\hat{\mathrm{x}} \pm \sin\left(K\cdot R_{\alpha,\mathrm{j}} + \omega_{K_1}\mathrm{t}\right)\hat{\mathrm{y}}$.

Supported by our experimental findings and the group theory analysis below, we will focus in this work on the modes K_{1-3}. Supplementary Figure 6a shows the atomic displacements that correspond to low- and high- energy phonon modes with symmetry K_1. The difference between the two is the phase difference in the circular motion of Se and W atoms, leading to mitigated deviation of the bond lengths from equilibrium in the low energy case. Supplementary Figure 6b shows the atomic displacement that corresponds to a phonon mode with symmetry K_3. Here, only Se atoms go through in-plane circular motion around their equilibrium positions and the phonon energy is somewhere between that of the low- and high-energy phonon modes with symmetry K_1.

Supplementary Figure 6c shows the atomic displacements that correspond to low- and high-energy phonon modes with symmetry K_2. Here, the transition-metal atoms go through in-plane circular motion around their equilibrium positions, while chalcogen atoms vibrate in opposite directions along the out-of-plane axis. As shown in the figure, the difference between the low and high energy modes is the relative motion of the chalcogen and transition-metal atoms. Namely, the low energy mode corresponds to the case that when the transition-metal atoms move toward the

chalcogen atoms, the chalcogen atoms move away from the mid-plane and vice versa. This combined motion keeps the bond length closer to its equilibrium value, and hence to lower phonon energy. Conversely, the high energy mode leads to stronger deviations from equilibrium because when the transition-metal atoms move toward the chalcogen atoms, the chalcogen atoms move closer to the mid-plane and vice versa.

Finally, Supplementary Figure 5b shows that the branch extensions of the ZA and TA modes anti-cross close to the K-point. This anti-crossing is not seen in the calculation of monolayer MoS_2. The result is that the mode K_6 has the lowest energy in monolayer MoS_2, while K_2 has the lowest energy in monolayer WeS_2.

Band-edge electronic states and selection rules for intervalley transitions

Next, we derive selection rules for intervalley transitions of electrons and holes. We first note that $K = 4\pi/3a$ is not only the wavenumber that connects each of the zone-edge $\pm K$-points with the zone center, but it is also the wavenumber needed to connect the zone-edge $+K$ and $-K$ points. In other words, the conservation of crystal momentum due to intervalley transitions of electrons (or holes) states at the $\pm K$ points is mediated through the zone-edge K-point phonons. We use Supplementary Table 3 to find the transformation properties of the zone-edge electronic states along with the ensuing selection rules for phonon-induced intervalley transitions of electrons and holes[11]. Starting with holes states, the transformation properties of their wavefunctions at the valence-band edge states are captured by the IRs K_7 and K_8. As shown in Supplementary Figure 5c, the band-edge hole state at the top $-K$ ($+K$) valley transforms like the IR K_7 (K_8), and vice versa for the bottom valleys. This behavior is a consequence of time-reversal symmetry and the complex conjugation of the IRs K_7 and K_8. Accordingly, a spin conserving intervalley scattering between the edge states involves the selection rule[11]

$$(K_7^* \times K_7)^* = (K_8^* \times K_8)^* = K_1\,, \qquad \text{(S3)}$$

where the first term corresponds to hole transition between the top valley in $-K$ and the bottom one in $+K$, while the second term corresponds to hole transition between the bottom valley in $-K$ and the top one in $+K$. This selection rule implies that the phonon mode K_1 is the dominant mechanism for intervalley transitions in the valence band.

The case of conduction-band electrons is somewhat more subtle. As shown in Supplementary Figure 5c, the band-edge electron state at the bottom (top) $-K$ valley transforms like K_9 (K_{11}), while the respective complex conjugate IRs at the $+K$ valleys transforms like K_{10} (K_{12}). The selection rule in this case reads[11]

$$(K_{12}^* \times K_9)^* = (K_{10}^* \times K_{11})^* = K_3\,, \qquad \text{(S4)}$$

implying that the phonon mode K_3 is the dominant mechanism for intervalley transitions in the conduction band.

Supplementary Note4. Qualitative comparison between theory and experiment

Analyzing the experimental findings of this work, we find very good agreement with the above theoretical analysis and previous predictions[11,12]. For example, Fig. 4 shows two phonon replicas of the indirect (intervalley) exciton, I^0, whose energies match the calculated values of the phonons modes K_1 (17 meV) and K_3 (26 meV) in Supplementary Figure 5b. These modes are suggested by

the selection rules in Eqs. (S3) and (S4), and they are supported by the cross-polarized emitted light from the phonon replica $I^0_{K_1}$ versus the co-polarized emitted light from the phonon replica $I^0_{K_3}$, as shown and analyzed in the main text. Similarly, we see that this behavior is consistent when dealing with dark trions states (Fig. 2 and Fig. 3).

One aspect of the experiment that can be further supported by theory is the observation that the PL phonon-assisted peaks associated with the mode K_3 are noticeably stronger in amplitude than those with K_1. Invoking second-order perturbation theory[14], the amplitude ratio of these peaks follows

$$\frac{\mathrm{A_{K_3}}}{\mathrm{A_{K_1}}} = \left|\frac{\mathcal{M}_{\mathcal{K}_3}}{\mathcal{M}_{\mathcal{K}_1}}\right|^2 \times \left|\frac{\mathrm{E_{D^\pm}} - \left(\mathrm{E_{X_B^\pm}} + \mathrm{E_{K_1}}\right)}{\mathrm{E_{D^\pm}} - \left(\mathrm{E_{X_A^\pm}} + \mathrm{E_{K_3}}\right)}\right|^2 . \qquad \text{(S5)}$$

$\mathcal{M}_{\mathcal{K}_3}$ and $\mathcal{M}_{\mathcal{K}_1}$ are the matrix elements for phonon-mediated intervalley transition in the conduction and valence bands, respectively. The other terms in Eq. (S5) are the energy differences between the initial state, here assumed to be the positive or negative dark trion ($D^\pm$), and the intermediate virtual states. For the case of intervalley hole transition, the energy of the intermediate state is that of the emitted phonon K_1 and the virtual type-B trion. For the case of intervalley electron transition, the energy of the intermediate state is that of the emitted phonon K_3 and the virtual bright type-A trion state (with singlet spin configuration of the same-sign charges).

The reason for the stronger signature of K_3 phonons compared with K_1 is the small spin-splitting energy of the conduction band compared with that of the valence-band, $\Delta_c \ll \Delta_v$. As a result, the energies of the initial and intermediate virtual states are relatively similar when the intermediate hole states are kept in the top valleys of the valence bands (i.e., when the electron goes through intervalley transition whereas the hole is a spectator). Substituting empirical values for the energies in monolayer WSe_2, $|E_{D^\pm} - (E_{X_A^\pm} + E_{K3})| \sim 60$ meV, and $|E_{D^\pm} - (E_{X_B^\pm} + E_{K1})| \sim \Delta_v \sim 400$ meV, we get that

$$\frac{\mathrm{A_{K_3}}}{\mathrm{A_{K_1}}} \sim 40 \left|\frac{\mathcal{M}_{\mathcal{K}_3}}{\mathcal{M}_{\mathcal{K}_1}}\right|^2 . \qquad \text{(S6)}$$

Our experimental results show that the amplitude ratio of the peaks associated with K_3 and K_1 is about 4, implying that $\mathcal{M}_{\mathcal{K}_1}$ should be about three times larger than $\mathcal{M}_{\mathcal{K}_3}$. This empirical analysis can be used to benchmark the results of future first-principles calculations of these matrix elements.

Supplementary Note5. The phonon replica K_2.

In addition to the replicas predicted by the previous group-theory analysis, we have observed a weak phonon replica in both the positive and negative dark trions that emerges ∼13 meV below the no-phonon dark trion lines (Fig.2 and Fig. 3). From the g-factor and polarization analysis, one can see that this replica, albeit weaker, shows the same characteristics of the replica K_3. Namely, it involves intervalley scattering of the electron component. A question remains regarding the physical origin of this replica. Inspecting the low-energy modes at the K-point, as shown in Supplementary Figure 5b, we can associate these peaks with either the lowest-energy mode K_2 or the second one K_6 (their calculated energies are 11.7 and 15.3 meV).

The mode K_6 involves out-of-plane vibration of the transition-metal atom, which breaks the mirror-inversion symmetry of the monolayer. In general, interactions of electrons (or holes) with such phonon modes can only couple to spin flips[11]. Unlike the zone-edge phonons with K_6 symmetry in which only W atoms vibrate in the out-of-plane direction, zone-edge phonons with K_2 symmetry involve counter motion of the Se atoms in the out-of-plane direction which retains the mirror inversion symmetry. Accordingly, the mode K_2 can induce a spin-conserving intervalley transition, albeit its amplitude is measurable only when the initial and final states are relatively far from being time reversal partners. In more detail, the selection rule in Eq. (S4) does not only mean that the amplitude of the matrix element $\mathcal{M}_{\mathcal{K}_3}$ is the dominant one for spin-conserving intervalley electron transitions, but that it is the only one for which the transition between the time-reversed $+K$ and $-K$ states does not vanish.

In conclusion, given that spin-conserving intervalley transitions consistently explain the recombination of electrons and holes from opposite valleys (see main text), we have attributed the low-energy phonon replica with K_2 rather than K_6.

Open discussion

1. Encapsulation by hBN breaks the mirror symmetry of the system. Thus, further experiments and theory analyses are needed to study if K_6 can also contribute to the low-energy phonon replica.
2. First-principle calculation of the matrix elements for spin-conserving intervalley transitions is needed for each of the nine zone-edge phonons. This calculation can be used to evaluate the ratio between the various phonon replicas, and explain the following question: why the phonon replicas K_1 and K_2 are observed only with the low-energy modes? For example, the selection rule in Eq. (S3) does not discern between the low- and high energy modes of K_1 (whose calculated values are 17 and 30 meV). Calculating the matrix elements should shed light on this question.
3. The localization of excitons (and trions) next to defects can enhance their coupling to phonons. Furthermore, defects alleviate the crystal symmetry and enable scattering of exciton complexes with phonon modes other than K_3 and K_1. As such, localization can explain the observation of the low-energy phonon replica that we have associated to the phonon mode K_2. Further experiments and theory analyses are needed to study the effect of localization on the phonon replica.

Supplementary Note6. Fine structure of the $D^0_{\Gamma_5}$.

Short-range exchange interaction between the electron and hole is predicted to lift the double degeneracy of D^0, the dark exciton[12]. Symmetry analysis further shows that the lower energy branch is strictly forbidden whereas the higher energy branch has an out of plane dipole and can give in-plane emission. As shown in Fig.4b, D^0 exhibits fine structure, displaying a finite zero field energy splitting, consistent with the previous report.[7]

Remarkably, we also observe the fine structure of the Γ_5 phonon replica of dark exciton, $D^0_{\Gamma_5}$. In the cross-polarized PL spectrum in Supplementary Figure 9a, $D^0_{\Gamma_5}$ shows an energy splitting of 0.6 meV. Magneto-PL spectroscopy further confirms the zero-field energy splitting (Supplementary Figure 9c) and reveals the hybridized nature of the two branches at low field. In contrast to the dark exciton D^0, which does not show circular polarization, $D^0_{\Gamma_5}$ becomes fully

circularly polarized at high field. This shows that whereas the lower energy branch of D^0 is strictly forbidden by symmetry, its Γ_5 phonon replica is allowed due to finite coupling to the bright exciton X^0.

This fine feature can also be well captured by the group theory analysis presented in SI-4. The dark (lower energy) and semi-dark (higher energy) branch of dark exciton D^0 can be represented by irreducible representation Γ_3 and Γ_4 respectively, whereas the bright exciton X^0 can be represented by Γ_6.[11] The selection rule Eq.(S1) then naturally allows the two dark excitons to be coupled to X^0 through a zone center Γ_5 phonon, leading to the observed fine structure of $D^0_{\Gamma_5}$.

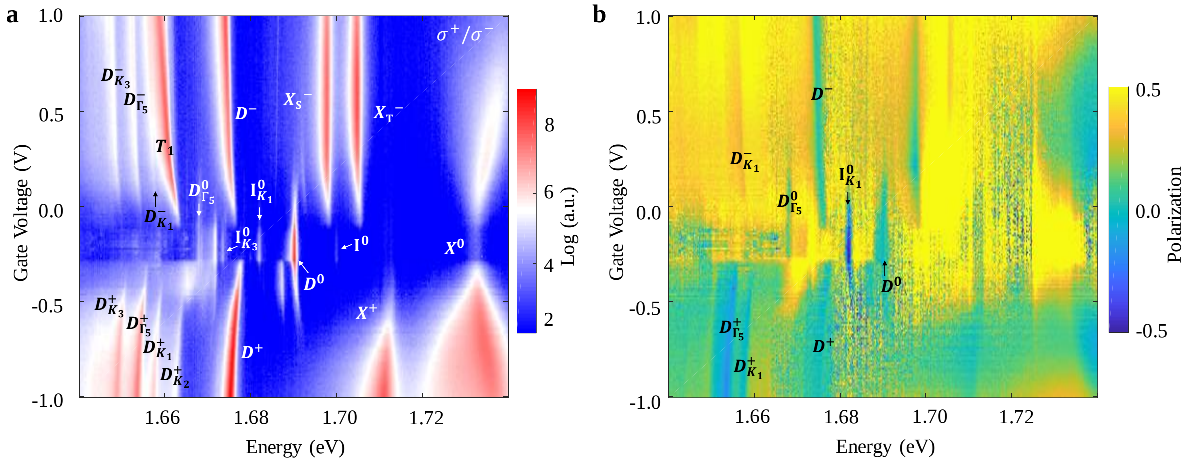

Supplementary Figure1| Gate dependent photoluminescence. a, PL intensity plot as a function of gate voltage and photon energy, from the same device as shown in Fig. 1c. The excitation and detection are cross circularly polarized (σ^+ excitation and σ^- detection). We marked the excitonic states that have been identified in this paper as well as previously reported. **b,** The degree of circular of polarization as a function of gate voltage. D^-, D^0 and D^+ have negligible circular polarization due to their out of plane dipole moment. $I^0_{K_1}$ and $D^+_{\Gamma_5}$ stand out for their obvious cross polarization.

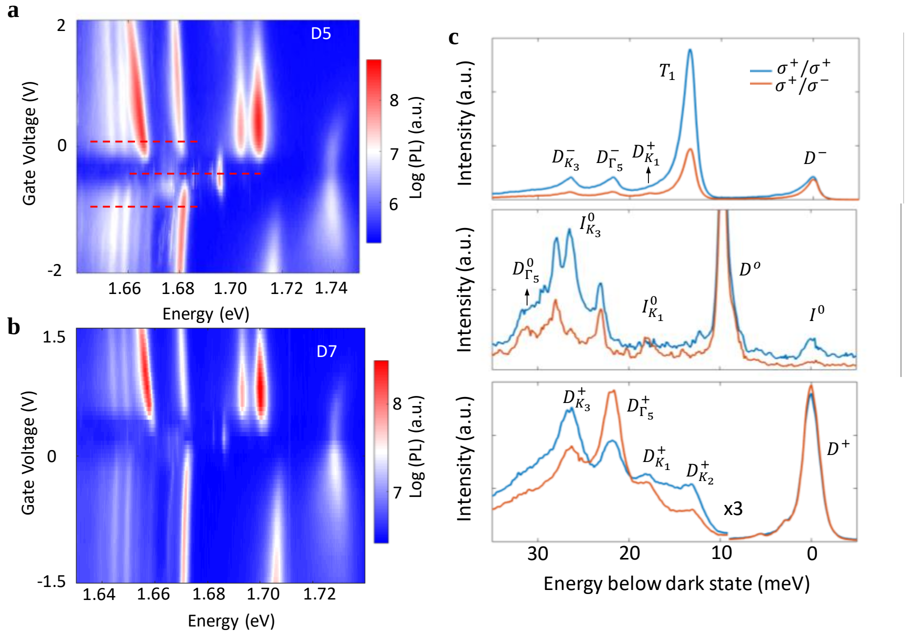

Supplementary Figure2| Reproducible gate dependent spectrum. a,b, Gate dependent PL spectrum in additional devices. Both devices show similar spectrum as in Fig. 1c, with well resolved valley phonon replicas. **c,** Circular polarization resolved PL in MD 5, at selective gate voltages indicated by the red lines with electron doping, neutral regime and hole doping from top to bottom. Valley phonon replicas and the dark excitonic states are labeled for clarity. The photon energy of the spectrum is relative to the corresponding indirect exciton (I^0) or dark trions ($D^{\pm}$).

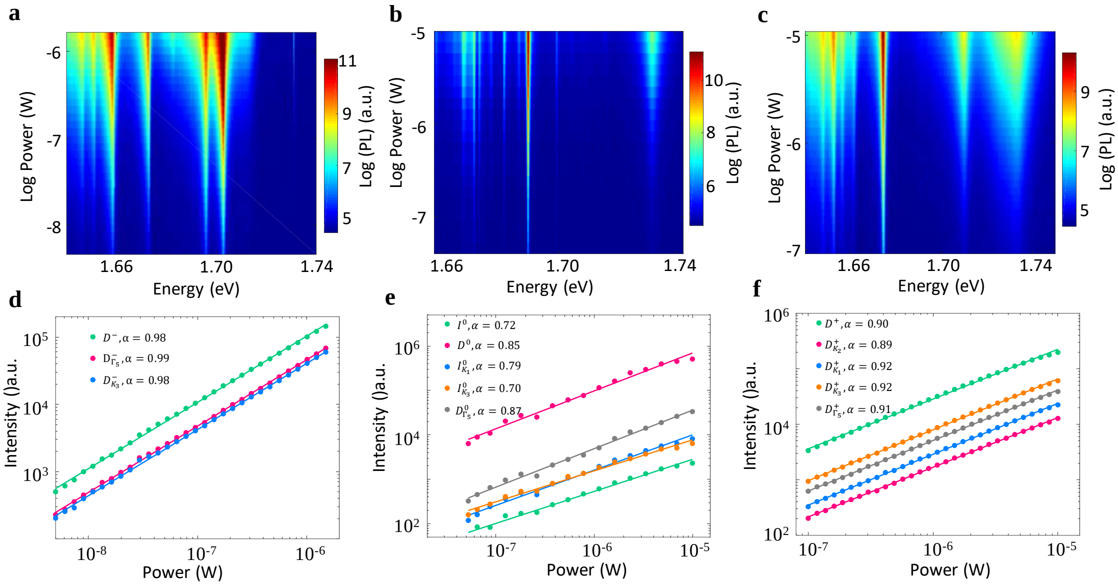

Supplementary Figure3| Power dependence of valley phonon-assisted emission. a-c, PL intensity as a function of pumping power, at electron doping, neutral regime and hole doping from left to right. **d-f,** Extracted intensity of dark states and their phonon replicas as a function of pumping power. The solid lines are power law fitting. For each dark state, the corresponding power dependence of valley phonon replicas are all similar. This further supports their valley assisted emission from the same dark state.

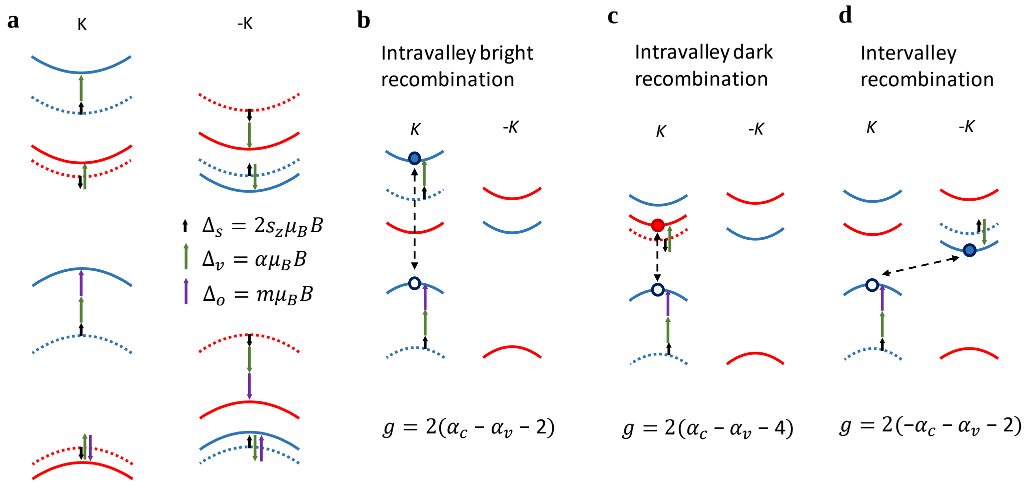

Supplementary Figure4| Effective Landé g-factor of excitonic states with different spin-valley configurations. a, Band diagram at $\pm K$ valley, showing three main contributions to Zeeman shifts: black for spin, green for valley magnetic moment, and purple for atomic orbital magnetic moment. **b-d,** Different spin-valley configuration of the recombined electron-hole pair, showing intravalley bright exciton recombination, intravalley dark exciton recombination, and intervalley recombination. Their corresponding effective g-factors are indicated at the bottom.

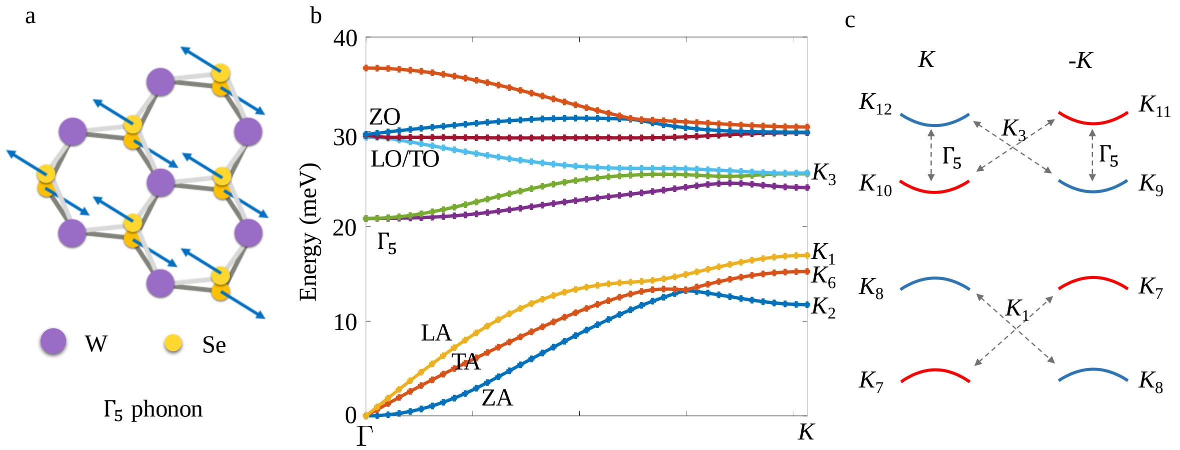

Supplementary Figure5| Phonon modes and selection rules. a, Atomic displacement of the phonon mode Γ_5. **b,** The phonon dispersion of monolayer WSe_2 along the axis between the high-symmetry Γ and *K* points. Zone-center and pertinent zone-edge phonon modes are indicated (symmetries and atomic displacements of the zone-edge phonon modes are analyzed in Supplementary Table 3 and Supplementary Figure 6). **c,** Scheme of low-energy valleys in the conduction and valence bands. Spin-flip intravalley transitions in the conduction band are mediated by the phonon-mode Γ_5, while spin-conserving intervalley transitions in the conduction (valence) bands are mediated by the phonon mode K_3 (K_1). Phonon-induced intervalley spin-flip scattering is relatively weak (the transition matrix element between time-reversed states vanishes).

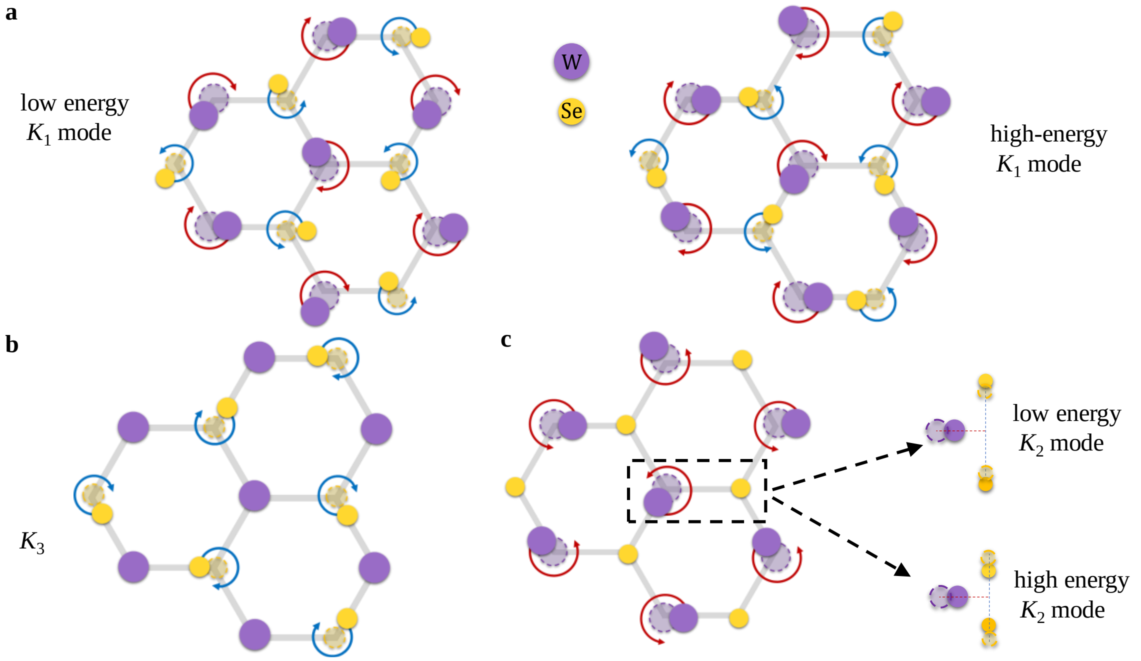

Supplementary Figure6| Atomic motions of Valley phonons. a-c, Atomic displacement of the valley phonon mode K_1, K_3 and K_2. The curved arrows denote the in-plane circular motion of atoms around their equilibrium positions, denoted by faint circles. See text for further explanations.

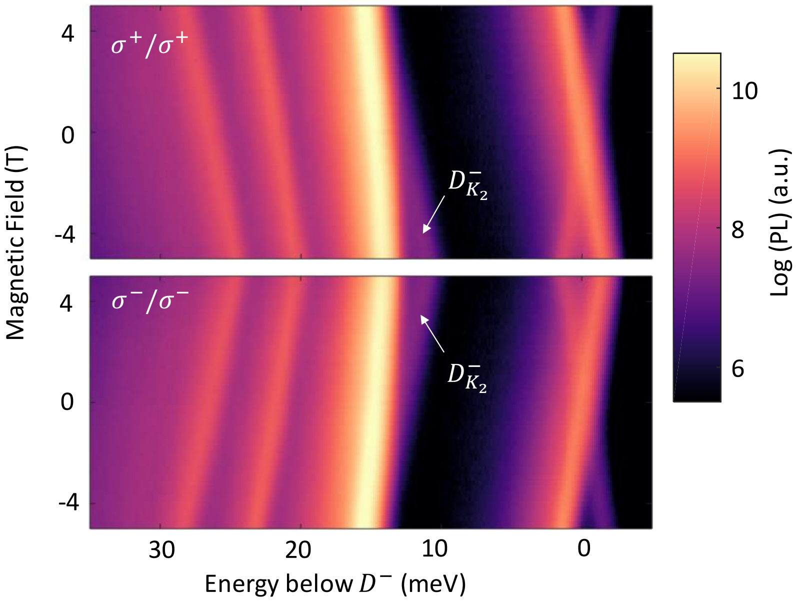

Supplementary Figure7| Magneto PL spectrum of the triplet emission pattern. Circular polarization resolved magneto PL spectrum of the triplet states with σ^+/σ^+(up) and σ^-/σ^-(down) polarized excitation and detection. The photon energy of the spectrum is offset with respect to D^- at zero field.

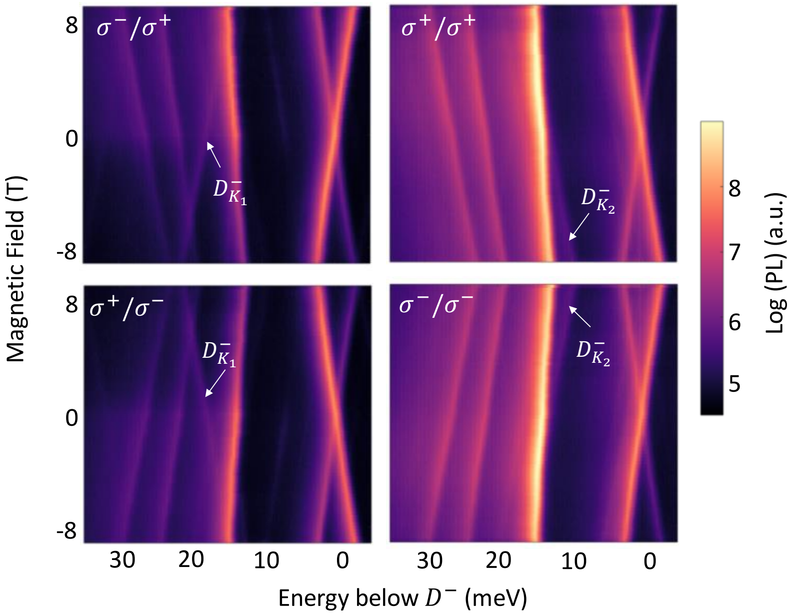

Supplementary Figure8| Magneto PL spectrum under resonant excitation of the bright neutral exciton. The sample is electron doped. The polarization configuration of excitation/collection is σ^-/σ^+ (top left), σ^+/σ^- (bottom left), σ^+/σ^+ (top right), σ^-/σ^- (bottom right). $D^-_{K_1}$ stands out in the cross polarized spectrum (left panels) with opposite Zeeman energy shift vs B compared with other valley phonon replicas. The faint peak on the higher energy shoulder of T_1 at high field corresponds to $D^-_{K_2}$.

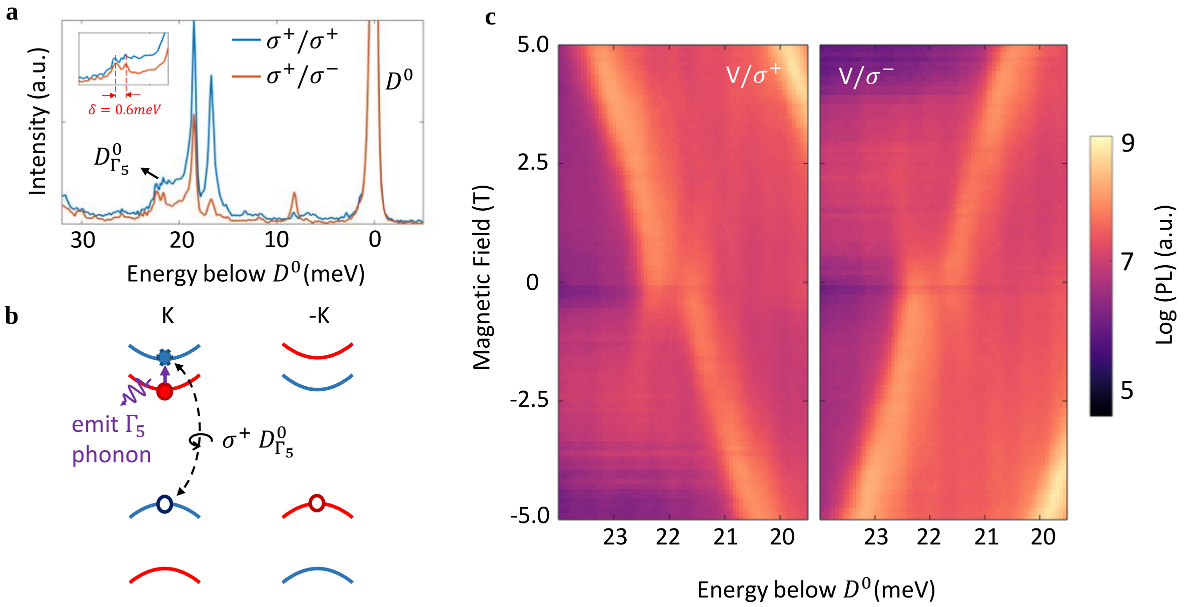

Supplementary Figure9 | Fine structure of the $D^0_{\Gamma_5}$. a, Circular polarization resolved PL at the charge neutral regime. The photon energy of the spectrum is shifted relative to the dark exciton D^0. The cross polarized PL shows the fine structure of $D^0_{\Gamma_5}$, with a splitting δ of 0.6meV in the peak energy position. **b,** Schematic of the light emission process of a σ^+ polarized $D^0_{\Gamma_5}$ photon. The electron of the dark exciton D^0 first experiences a virtual intravalley spin flip transition while emitting a Γ_5 phonon. The electron-hole pair then couples with the bright exciton X^0, recombines, and emits a σ^+ polarized photon. **c,** A Zoom in plot of magneto PL spectrum from Fig. 4b, focusing on $D^0_{\Gamma_5}$. The spectrum is taken with linearly polarized excitation, σ^+ (left) and σ^- polarized (right) collection. The photon energy of the spectrum is shifted relative the dark exciton D^0.

	**Bright state**	**Dark state**	$\boldsymbol{K_2}$ **replica**	$\boldsymbol{K_1}$ **replica**	$\boldsymbol{\Gamma_5}$ **replica**	$\boldsymbol{K_3}$ **replica**
**neutral regime**	-4.7	I^0 -12.5	/	12.0	/	-12.6
		D^0 -9.1	/	/	-9.8	/
**Hole doped:** $\boldsymbol{D^+}$	-2	-8.6	-13.0	12.2	-9.7	-13.4
**Electron doped:** $\boldsymbol{D^-}$	X_T^- -5.2	-9.5	-13.6	12.2	-9.9	-12.5
	X_S^+ -3.4					

Supplementary Table1| Landé effective g-factor of identified excitonic states. Effective g-factor of identified excitonic states in Supplementary Figure 1a. For readers' convenience, g-factor of dark states and their phonon replicas in Table 1 are listed here as well.

$\boldsymbol{D_{3h}}$		$\boldsymbol{E}$	$\boldsymbol{\overline{E}}$	$\boldsymbol{C_3^+C_3^-}$	$\boldsymbol{\overline{C}_3^+\overline{C}_3^-}$	$\boldsymbol{\sigma_h\overline{\sigma}_h}$	$\boldsymbol{S_3^+S_3^-}$	$\boldsymbol{\overline{S}_3^+\overline{S}_3^-}$	$\boldsymbol{C'_{2i}\overline{C}'_{2i}}$	$\boldsymbol{\sigma_{vi}\overline{\sigma}_{vi}}$
$\boldsymbol{A'_1}$	$\boldsymbol{\Gamma_1}$	1	1	1	1	1	1	1	1	1
$\boldsymbol{A'_2}$	$\boldsymbol{\Gamma_2}$	1	1	1	1	1	1	1	-1	-1
$\boldsymbol{A''_1}$	$\boldsymbol{\Gamma_3}$	1	1	1	1	-1	-1	-1	1	-1
$\boldsymbol{A''_2}$	$\boldsymbol{\Gamma_4}$	1	1	1	1	-1	-1	-1	-1	1
$\boldsymbol{E''}$	$\boldsymbol{\Gamma_5}$	2	2	-1	-1	-2	1	1	0	0
$\boldsymbol{E'}$	$\boldsymbol{\Gamma_6}$	2	2	-1	-1	2	-1	-1	0	0
$\boldsymbol{\overline{E}_1}$	$\boldsymbol{\Gamma_7}$	2	-2	1	-1	0	$\sqrt{3}$	$-\sqrt{3}$	0	0
$\boldsymbol{\overline{E}_2}$	$\boldsymbol{\Gamma_8}$	2	-2	1	-1	0	$-\sqrt{3}$	$\sqrt{3}$	0	0
$\boldsymbol{\overline{E}_3}$	$\boldsymbol{\Gamma_9}$	2	-2	2	2	0	0	0	0	0

Supplementary Table2| Character table of $\boldsymbol{D_{3h}}$ **point double-group.** It captures the symmetry properties of the Γ point in ML-TMDs. The *x*-axis is along zigzag edge direction and the *y*-axis is along

C_{3h}		E	C_3^+	C_3^-	σ_h	S_3^+	S_3^-	$\overline{E}$	$\overline{C_3^+}$	$\overline{C_3^-}$	$\overline{\sigma_h}$	$\overline{S_3^+}$	$\overline{S_3^-}$	Polarization vectors
A'	K_1	1	1	1	1	1	1	1	1	1	1	1	1	$M_{\parallel}(1,-i,0)$ $\pm X_{+,\parallel}(1,i,0)$
$^2E'$	K_2	1	ω	ω^*	1	ω	ω^*	1	ω	ω^*	1	ω	ω^*	$X_{-,Z} \pm M_- \parallel (1,i,0)$
$^1E'$	K_3	1	ω^*	ω	1	ω^*	ω	1	ω^*	ω	1	ω^*	ω	$X_{+,\parallel}(1,-i,0)$
A''	K_4	1	1	1	-1	-1	-1	1	1	1	-1	-1	-1	$X_{-,\parallel}(1,i,0)$
$^2E''$	K_5	1	ω	ω^*	-1	-ω	-ω^*	1	ω	ω^*	-1	-ω	-ω^*	$X_{+,z}$
$^1E''$	K_6	1	ω^*	ω	-1	-ω^*	-ω	1	ω^*	ω	-1	-ω^*	-ω	$M_z \pm X_{-,\parallel}(1,-i,0)$
$^1\overline{E}_3$	K_7	1	-ω	-ω^*	i	-$i\omega$	$i\omega^*$	-1	ω	ω^*	$-i$	$i\omega$	-$i\omega^*$	
$^2\overline{E}_3$	K_8	1	-ω^*	-ω	$-i$	$i\omega^*$	-$i\omega$	-1	ω^*	ω	i	-$i\omega^*$	$i\omega$	
$^2\overline{E}_2$	K_9	1	-ω	-ω^*	$-i$	$i\omega$	-$i\omega^*$	-1	ω	ω^*	i	-$i\omega$	$i\omega^*$	
$^1\overline{E}_2$	K_{10}	1	-ω^*	-ω	i	-$i\omega^*$	$i\omega$	-1	ω^*	ω	$-i$	$i\omega^*$	-$i\omega$	
$^1\overline{E}_1$	K_{11}	1	-1	-1	i	-i	i	-1	1	1	-i	i	-i	
$^2\overline{E}_1$	K_{12}	1	-1	-1	$-i$	i	$-i$	-1	1	1	i	$-i$	i	

Supplementary Table3| Character table of C_{3h} point double-group. This table captures the symmetry properties of the K point in ML-TMDs where $\omega = \exp(2\pi i/3)$. To prevent confusion with the notation in Supplementary Table 2, the Koster notation of the IRs is changed from Γ_i to K_i. The x-axis is along the zigzag edge direction and the y-axis is along the armchair direction.

Reference

1 Aivazian, G. *et al.* Magnetic control of valley pseudospin in monolayer WSe_2. *Nat. Phys.* **11**, 148 (2015).

2 Srivastava, A. *et al.* Valley Zeeman effect in elementary optical excitations of monolayer WSe_2. *Nat. Phys.* **11**, 141 (2015).

3 Li, Y. *et al.* Valley Splitting and Polarization by the Zeeman Effect in Monolayer $MoSe_2$. *Phys. Rev. Lett.* **113**, 266804 (2014).

4 MacNeill, D. *et al.* Breaking of Valley Degeneracy by Magnetic Field in Monolayer $MoSe_2$. *Phys. Rev. Lett.* **114**, 037401 (2015).

5 Xiao, D., Liu, G.-B., Feng, W., Xu, X. & Yao, W. Coupled Spin and Valley Physics in Monolayers of MoS_2 and Other Group-VI Dichalcogenides. *Phys. Rev. Lett.* **108**, 196802 (2012).

6 Xu, X. D., Yao, W., Xiao, D. & Heinz, T. F. Spin and pseudospins in layered transition metal dichalcogenides. *Nat. Phys.* **10**, 343-350 (2014).

7 Robert, C. *et al.* Fine structure and lifetime of dark excitons in transition metal dichalcogenide monolayers. *Phys Rev B* **96**, 155423 (2017).

8 Liu, E. *et al.* Chiral-phonon replicas of dark excitonic states in monolayer WSe_2. *arXiv preprint arXiv:1906.02323* (2019).

9 Li, Z. *et al.* Emerging photoluminescence from the dark-exciton phonon replica in monolayer WSe_2. *Nat. Commun.* **10**, 2469 (2019).

10 Bradley, C. & Cracknell, A. *The Mathematical Theory of Symmetry in Solids: Representation Theory for Point Groups and Space Groups*. (Oxford University Press, Oxford, 2009).

11 Song, Y. & Dery, H. Transport theory of monolayer transition-metal dichalcogenides through symmetry. *Phys. Rev. Lett.* **111**, 026601 (2013).

12 Dery, H. & Song, Y. Polarization analysis of excitons in monolayer and bilayer transition-metal dichalcogenides. *Phys Rev B* **92**, 125431 (2015).

13 Giannozzi, P. *et al.* QUANTUM ESPRESSO: a modular and open-source software project for quantum simulations of materials. *J. Phys. Condens. Matter* **21**, 395502 (2009).

14 Cardona, M. & Peter, Y. Y. *Fundamentals of Semiconductors*. (Springer, Berlin, 2005).
